# (*Z*)-(1,2-Dichloro­vin­yl)diphenyl­phosphine oxide

**DOI:** 10.1107/S1600536811030765

**Published:** 2011-08-06

**Authors:** Jing-Ya Ma, Qing-Qin Feng, Ming-Shu Wu

**Affiliations:** a College of Chemistry & Chemical Engineering, Hainan Normal University, Haikou 571158, People’s Republic of China; b College of Chemistry & Chemical Engineering, Anyang Normal University, Anyang 455000, Henan, People’s Republic of China

## Abstract

The title compound, C_14_H_11_Cl_2_OP, was synthesized by the reaction of diphenyl­phosphine oxide with 1,2-dichloro­ethyne under CuI catalysis. The reaction provided the *Z* isomer regioselectively. Two O—P—C bond angles [114.3 (1) and 112.5 (1)°] are significantly larger than the C—P—C [107.7 (1), 105.6 (1) and 106.6 (1)°] and another O—P—C angle [109.5 (1)°], indicating significant distortion of the tetra­hedral configuration of the P atom. In the crystal, mol­ecules are linked by weak inter­molecular C—H⋯O hydrogen bonds into centrosymmetric dimers, which are connected by further C—H⋯O inter­actions into chains along [101].

## Related literature

For the anti­microbial, insecticidal and anti-inflammatory activity of alkenylphosphine oxides, see: Haynes *et al.* (1989 ▶, 1991 ▶); Shi *et al.* (2000 ▶); Taylor *et al.* (2006 ▶); Rahman *et al.* (2000 ▶). For their use as inter­mediates in the preparation of some palladium catalysts, see: Inoue *et al.* (2002 ▶). Nucleophiles, such as amines (Rahman *et al.*, 2000 ▶, 2004 ▶), phosphines (Barbaro *et al.*, 2002 ▶; Alajarin *et al.*, 2004 ▶; Han & Zhao, 2005 ▶) and carbanion species readily add to the olefinic bond in alkenylphosphine oxides to give useful bifunctional adducts. 
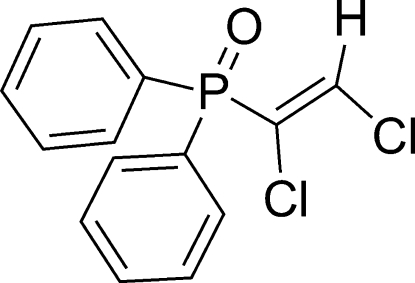


## Experimental

### 

#### Crystal data

C_14_H_11_Cl_2_OP
*M* *_r_* = 297.10
Monoclinic, 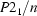

*a* = 12.0621 (11) Å
*b* = 7.9521 (8) Å
*c* = 14.9913 (15) Å
β = 102.858 (1)°
*V* = 1401.9 (2) Å^3^
*Z* = 4
Mo *K*α radiation
μ = 0.56 mm^−1^
*T* = 298 K
0.45 × 0.40 × 0.32 mm

#### Data collection

Bruker APEXII CCD area-detector diffractometer
Absorption correction: multi-scan (*SADABS*; Sheldrick, 2008 ▶) *T*_min_ = 0.786, *T*_max_ = 0.841
6788 measured reflections
2475 independent reflections
2009 reflections with *I* > 2σ(*I*)
*R*_int_ = 0.022

#### Refinement

*R*[*F* ^2^ > 2σ(*F* ^2^)] = 0.035
*wR*(*F* ^2^) = 0.094
*S* = 1.08
2475 reflections
163 parameters
H-atom parameters constrained
Δρ_max_ = 0.28 e Å^−3^
Δρ_min_ = −0.37 e Å^−3^

### 

Data collection: *APEX2* (Bruker, 2002 ▶); cell refinement: *SAINT* (Bruker, 2002 ▶); data reduction: *SAINT*; program(s) used to solve structure: *SHELXS97* (Sheldrick, 2008 ▶); program(s) used to refine structure: *SHELXL97* (Sheldrick, 2008 ▶); molecular graphics: *SHELXTL* (Sheldrick, 2008 ▶); software used to prepare material for publication: *SHELXTL*.

## Supplementary Material

Crystal structure: contains datablock(s) I, global. DOI: 10.1107/S1600536811030765/ld2021sup1.cif

Structure factors: contains datablock(s) I. DOI: 10.1107/S1600536811030765/ld2021Isup2.hkl

Supplementary material file. DOI: 10.1107/S1600536811030765/ld2021Isup3.cml

Additional supplementary materials:  crystallographic information; 3D view; checkCIF report

## Figures and Tables

**Table 1:** Hydrogen-bond geometry (Å, °)

*D*—H⋯*A*	*D*—H	H⋯*A*	*D*⋯*A*	*D*—H⋯*A*
C2—H2⋯O1^i^	0.93	2.51	3.138 (3)	125
C8—H8⋯O1^ii^	0.93	2.57	3.344 (4)	141

Symmetry codes: (i) 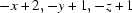; (ii) 
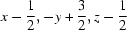
.

**Fig. 1.**
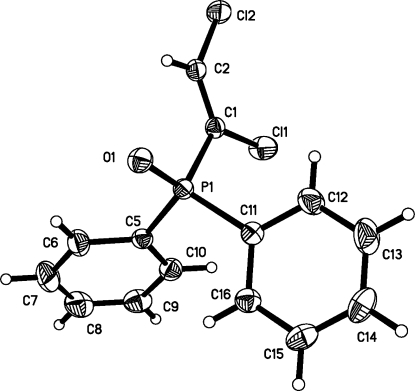
The molecular structure of the title compound, with atom labels and 50% probability displacement ellipsoids for non-H atoms.

**Fig. 2.**
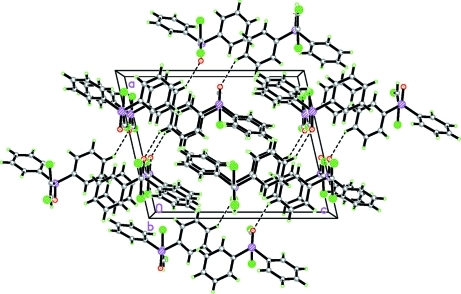
Crystal packing of the title compound, viewed down the b direction Intermolecular hydrogen bonds are shown as dashed lines.

## supplementary crystallographic information

### Comment

Alkenylphosphine oxides have attracted much attention because they are used as biologically active compounds (Haynes *et al.*, 1989; Haynes *et al.*, 1991; Shi *et al.*, 2000), and are the key intermediates for preparation of some palladium catalysts (Inoue *et al.*,2002). Nucleophiles, such as amines (Rahman *et al.*, 2000; Rahman *et al.*, 2004), phosphines (Barbaro *et al.*, 2002; Alajarin *et al.*, 2004; Han & Zhao, 2005) and carbanion species readily add to the olefinic bond in alkenylphosphine oxides to give useful bifunctional adducts. In order to further confirm stereostructure and structure-activity relationship of alkenylphosphine oxides, we performed the synthesis of the title compound by addition reaction of the diphenylphosphine oxide with 1,2-dichloroethyne under catalysis of commercially available CuI at room temperature. The reaction provided (Z)-(1,2-dichlorovinyl)diphenylphosphine oxide regioselectively. The study of the crystal structure of the title compound was commenced to establish its structural features that can be helpful for its practical applications. In the title molecule (Fig. 1), the bond angles O(1)–P(1)–C(11)(114.31 (10)°) and O(1)–P(1)–C(5)(112.55 (10)°) are significantly larger than C(11)–P(1)–C(5)(107.75 (10)°), O(1)–P(1)–C(1)(109.54 (10)°), C(11)–P(1)–C(1)(105.58 (10)°), and C(5)–P(1)–C(1) (106.62 (10)°), indicating that *sp^3^*-hybridized P atom adopts distorted tetrahedral configuration. In the crystal, molecules are linked by weak intermolecular C-H···O hydrogen bonds in centrosymmetric dimers further connected in chains along [1 0 1].

### Experimental

To a stirred solution of 1,2-dichloroethyne (1.88 g, 20 mmol) in anhydrous ether (40 ml), a solution of diphenylphosphine oxide (2.23g, 12mmol) in ether (10 ml) was added dropwise in the presence of CuI (0.229g, 1.2 mmol) at room temperature in nitrogen atmosphere for 4h. After completion of the reaction as indicated by thin-layer chromatography, the mixture was filtered and rinsed with ethyl acetate. The organic layer was washed with brine and dried over MgSO_4_. The title product was obtained by crystallization as colourless solid. The product obtained was purified by flash chromatograghy. Single crystals of the title compound suitable for single-crystal X-ray analysis were obtained by recrystallization from ether.

### Refinement

The H atoms were positioned geometrically and refined using a riding model with C—H = 0.93-0.98 Å and with *U*_iso_(H)= 1.2*U*_eq_(C).

### Crystal data

**Table:**

C_14_H_11_Cl_2_OP	*F*(000) = 608
*M**_r_* = 297.10	*D*_x_ = 1.408 Mg m^−^^3^
Monoclinic, *P*2_1_/*n*	Mo *K*α radiation, λ = 0.71073 Å
*a* = 12.0621 (11) Å	Cell parameters from 3636 reflections
*b* = 7.9521 (8) Å	θ = 2.5–28.0°
*c* = 14.9913 (15) Å	µ = 0.56 mm^−^^1^
β = 102.858 (1)°	*T* = 298 K
*V* = 1401.9 (2) Å^3^	Prism, colourless
*Z* = 4	0.45 × 0.40 × 0.32 mm

### Data collection

**Table:**

Bruker APEXII CCD area-detector diffractometer	2475 independent reflections
Radiation source: fine-focus sealed tube	2009 reflections with *I* > 2σ(*I*)
graphite	*R*_int_ = 0.022
φ and ω scans	θ_max_ = 25.0°, θ_min_ = 2.5°
Absorption correction: multi-scan (*SADABS*; Sheldrick, 2008)	*h* = −14→11
*T*_min_ = 0.786, *T*_max_ = 0.841	*k* = −9→9
6788 measured reflections	*l* = −12→17

### Refinement

**Table:**

Refinement on *F*^2^	Primary atom site location: structure-invariant direct methods
Least-squares matrix: full	Secondary atom site location: difference Fourier map
*R*[*F*^2^ > 2σ(*F*^2^)] = 0.035	Hydrogen site location: inferred from neighbouring sites
*wR*(*F*^2^) = 0.094	H-atom parameters constrained
*S* = 1.08	*w* = 1/[σ^2^(*F*_o_^2^) + (0.0395*P*)^2^ + 0.729*P*] where *P* = (*F*_o_^2^ + 2*F*_c_^2^)/3
2475 reflections	(Δ/σ)_max_ = 0.001
163 parameters	Δρ_max_ = 0.28 e Å^−^^3^
0 restraints	Δρ_min_ = −0.37 e Å^−^^3^

### Special details

**Table:**

Geometry. All esds (except the esd in the dihedral angle between two l.s. planes) are estimated using the full covariance matrix. The cell esds are taken into account individually in the estimation of esds in distances, angles and torsion angles; correlations between esds in cell parameters are only used when they are defined by crystal symmetry. An approximate (isotropic) treatment of cell esds is used for estimating esds involving l.s. planes.
Refinement. Refinement of F^2^ against ALL reflections. The weighted R-factor wR and goodness of fit S are based on F^2^, conventional R-factors R are based on F, with F set to zero for negative F^2^. The threshold expression of F^2^ > 2sigma(F^2^) is used only for calculating R-factors(gt) etc. and is not relevant to the choice of reflections for refinement. R-factors based on F^2^ are statistically about twice as large as those based on F, and R- factors based on ALL data will be even larger.

### Fractional atomic coordinates and isotropic or equivalent isotropic displacement parameters (Å^2^)

**Table:**

	*x*	*y*	*z*	*U*_iso_*/*U*_eq_	
Cl1	0.64091 (5)	0.30633 (8)	0.48897 (6)	0.0610 (2)	
Cl2	0.87750 (6)	0.09961 (8)	0.52709 (5)	0.0558 (2)	
O1	0.90014 (13)	0.6840 (2)	0.53670 (11)	0.0441 (4)	
P1	0.77986 (4)	0.62950 (7)	0.51027 (4)	0.03243 (16)	
C1	0.77287 (18)	0.4017 (3)	0.51179 (15)	0.0340 (5)	
C2	0.8685 (2)	0.3151 (3)	0.52622 (15)	0.0386 (5)	
H2	0.9363	0.3754	0.5367	0.046*	
C5	0.71157 (19)	0.6946 (3)	0.39607 (15)	0.0358 (5)	
C6	0.7790 (2)	0.7721 (3)	0.34425 (18)	0.0528 (7)	
H6	0.8557	0.7909	0.3694	0.063*	
C7	0.7327 (3)	0.8213 (4)	0.2555 (2)	0.0726 (9)	
H7	0.7782	0.8733	0.2211	0.087*	
C8	0.6190 (3)	0.7938 (4)	0.2178 (2)	0.0693 (9)	
H8	0.5883	0.8266	0.1579	0.083*	
C9	0.5516 (2)	0.7187 (4)	0.26807 (19)	0.0580 (7)	
H9	0.4749	0.7011	0.2422	0.070*	
C10	0.5964 (2)	0.6681 (3)	0.35770 (17)	0.0462 (6)	
H10	0.5500	0.6171	0.3918	0.055*	
C11	0.69328 (18)	0.7001 (3)	0.58622 (15)	0.0357 (5)	
C12	0.6898 (3)	0.6104 (3)	0.66513 (18)	0.0570 (7)	
H12	0.7270	0.5076	0.6761	0.068*	
C13	0.6313 (3)	0.6733 (4)	0.7274 (2)	0.0720 (9)	
H13	0.6287	0.6122	0.7798	0.086*	
C14	0.5770 (3)	0.8255 (4)	0.7119 (2)	0.0646 (8)	
H14	0.5375	0.8672	0.7538	0.078*	
C15	0.5807 (2)	0.9163 (4)	0.6352 (2)	0.0574 (7)	
H15	0.5446	1.0202	0.6255	0.069*	
C16	0.6382 (2)	0.8539 (3)	0.57215 (17)	0.0450 (6)	
H16	0.6399	0.9157	0.5198	0.054*	

### Atomic displacement parameters (Å^2^)

**Table:**

	*U*^11^	*U*^22^	*U*^33^	*U*^12^	*U*^13^	*U*^23^
Cl1	0.0402 (4)	0.0432 (4)	0.0962 (6)	−0.0081 (3)	0.0078 (3)	0.0024 (3)
Cl2	0.0635 (4)	0.0370 (3)	0.0740 (5)	0.0102 (3)	0.0305 (4)	0.0031 (3)
O1	0.0339 (8)	0.0455 (9)	0.0512 (10)	−0.0043 (7)	0.0057 (7)	−0.0002 (8)
P1	0.0304 (3)	0.0327 (3)	0.0339 (3)	−0.0002 (2)	0.0064 (2)	0.0015 (2)
C1	0.0356 (12)	0.0325 (11)	0.0348 (12)	−0.0024 (9)	0.0100 (9)	−0.0005 (9)
C2	0.0436 (13)	0.0330 (12)	0.0430 (14)	0.0015 (10)	0.0181 (11)	0.0019 (10)
C5	0.0396 (12)	0.0333 (11)	0.0351 (12)	0.0037 (9)	0.0095 (10)	0.0002 (9)
C6	0.0531 (15)	0.0594 (16)	0.0476 (15)	−0.0026 (13)	0.0152 (12)	0.0103 (13)
C7	0.088 (2)	0.086 (2)	0.0489 (18)	0.0013 (18)	0.0252 (16)	0.0236 (16)
C8	0.088 (2)	0.078 (2)	0.0381 (16)	0.0183 (18)	0.0070 (16)	0.0105 (15)
C9	0.0536 (16)	0.0668 (18)	0.0458 (16)	0.0115 (14)	−0.0053 (13)	−0.0015 (14)
C10	0.0438 (14)	0.0517 (14)	0.0422 (14)	0.0045 (11)	0.0078 (11)	0.0023 (12)
C11	0.0358 (12)	0.0386 (12)	0.0313 (12)	−0.0010 (9)	0.0046 (9)	−0.0027 (10)
C12	0.0811 (19)	0.0525 (15)	0.0396 (14)	0.0128 (14)	0.0181 (13)	0.0058 (12)
C13	0.104 (3)	0.078 (2)	0.0404 (16)	−0.0032 (19)	0.0308 (17)	−0.0010 (15)
C14	0.0630 (18)	0.082 (2)	0.0546 (18)	−0.0031 (16)	0.0253 (14)	−0.0256 (16)
C15	0.0553 (16)	0.0576 (16)	0.0591 (18)	0.0106 (13)	0.0122 (14)	−0.0158 (14)
C16	0.0478 (14)	0.0424 (13)	0.0443 (14)	0.0025 (11)	0.0089 (11)	−0.0008 (11)

### Geometric parameters (Å, °)

**Table:**

Cl1—C1	1.727 (2)	C8—H8	0.9300
Cl2—C2	1.717 (2)	C9—C10	1.391 (4)
O1—P1	1.4813 (16)	C9—H9	0.9300
P1—C11	1.798 (2)	C10—H10	0.9300
P1—C5	1.803 (2)	C11—C16	1.385 (3)
P1—C1	1.814 (2)	C11—C12	1.390 (3)
C1—C2	1.319 (3)	C12—C13	1.382 (4)
C2—H2	0.9300	C12—H12	0.9300
C5—C6	1.388 (3)	C13—C14	1.371 (5)
C5—C10	1.397 (3)	C13—H13	0.9300
C6—C7	1.380 (4)	C14—C15	1.366 (4)
C6—H6	0.9300	C14—H14	0.9300
C7—C8	1.379 (5)	C15—C16	1.384 (4)
C7—H7	0.9300	C15—H15	0.9300
C8—C9	1.364 (4)	C16—H16	0.9300
			
O1—P1—C11	114.31 (10)	C8—C9—C10	120.6 (3)
O1—P1—C5	112.55 (10)	C8—C9—H9	119.7
C11—P1—C5	107.75 (10)	C10—C9—H9	119.7
O1—P1—C1	109.54 (10)	C9—C10—C5	119.4 (2)
C11—P1—C1	105.58 (10)	C9—C10—H10	120.3
C5—P1—C1	106.62 (10)	C5—C10—H10	120.3
C2—C1—Cl1	122.49 (18)	C16—C11—C12	118.6 (2)
C2—C1—P1	118.80 (17)	C16—C11—P1	120.36 (18)
Cl1—C1—P1	118.64 (12)	C12—C11—P1	120.72 (18)
C1—C2—Cl2	124.99 (19)	C13—C12—C11	120.4 (3)
C1—C2—H2	117.5	C13—C12—H12	119.8
Cl2—C2—H2	117.5	C11—C12—H12	119.8
C6—C5—C10	119.3 (2)	C14—C13—C12	120.1 (3)
C6—C5—P1	117.27 (18)	C14—C13—H13	120.0
C10—C5—P1	123.39 (18)	C12—C13—H13	120.0
C7—C6—C5	120.2 (3)	C15—C14—C13	120.4 (3)
C7—C6—H6	119.9	C15—C14—H14	119.8
C5—C6—H6	119.9	C13—C14—H14	119.8
C8—C7—C6	120.2 (3)	C14—C15—C16	120.0 (3)
C8—C7—H7	119.9	C14—C15—H15	120.0
C6—C7—H7	119.9	C16—C15—H15	120.0
C9—C8—C7	120.2 (3)	C15—C16—C11	120.6 (2)
C9—C8—H8	119.9	C15—C16—H16	119.7
C7—C8—H8	119.9	C11—C16—H16	119.7
			
O1—P1—C1—C2	−6.2 (2)	C7—C8—C9—C10	−0.3 (5)
C11—P1—C1—C2	−129.75 (19)	C8—C9—C10—C5	−0.1 (4)
C5—P1—C1—C2	115.8 (2)	C6—C5—C10—C9	0.5 (4)
O1—P1—C1—Cl1	176.78 (12)	P1—C5—C10—C9	−178.29 (19)
C11—P1—C1—Cl1	53.24 (16)	O1—P1—C11—C16	88.7 (2)
C5—P1—C1—Cl1	−61.18 (16)	C5—P1—C11—C16	−37.2 (2)
Cl1—C1—C2—Cl2	−1.4 (3)	C1—P1—C11—C16	−150.85 (19)
P1—C1—C2—Cl2	−178.31 (13)	O1—P1—C11—C12	−84.5 (2)
O1—P1—C5—C6	5.5 (2)	C5—P1—C11—C12	149.6 (2)
C11—P1—C5—C6	132.48 (19)	C1—P1—C11—C12	35.9 (2)
C1—P1—C5—C6	−114.6 (2)	C16—C11—C12—C13	0.8 (4)
O1—P1—C5—C10	−175.63 (18)	P1—C11—C12—C13	174.1 (2)
C11—P1—C5—C10	−48.7 (2)	C11—C12—C13—C14	−0.6 (5)
C1—P1—C5—C10	64.3 (2)	C12—C13—C14—C15	−0.2 (5)
C10—C5—C6—C7	−0.4 (4)	C13—C14—C15—C16	0.8 (5)
P1—C5—C6—C7	178.4 (2)	C14—C15—C16—C11	−0.6 (4)
C5—C6—C7—C8	0.0 (5)	C12—C11—C16—C15	−0.2 (4)
C6—C7—C8—C9	0.4 (5)	P1—C11—C16—C15	−173.6 (2)

### Hydrogen-bond geometry (Å, °)

**Table:**

*D*—H···*A*	*D*—H	H···*A*	*D*···*A*	*D*—H···*A*
C2—H2···O1^i^	0.93	2.51	3.138 (3)	125.
C8—H8···O1^ii^	0.93	2.57	3.344 (4)	141.

Symmetry codes: (i) −*x*+2, −*y*+1, −*z*+1; (ii) *x*−1/2, −*y*+3/2, *z*−1/2.
